# A geographic information system to study trauma epidemiology in India

**DOI:** 10.1186/1752-2897-1-3

**Published:** 2007-11-26

**Authors:** Vaibhav Bagaria, Saurabh Bagaria

**Affiliations:** 1 NIIDAAN Ortho Centre, Nagpur, India; 2 Map Info, Noida, India

## Abstract

**Background:**

Geographic Information Systems (GIS) describe the topography and chronology of events in a defined vector space. GIS may also be used for an integrated analysis of environmental and road-related risk factors for traffic accidents.

**Methods:**

In a retrospective study, various features of 165 road crashes were linked to a GIS-generated digital map of an area close to a national highway in India. By overlay tools, clusters of accidents were identified, and color-coded according to accident mechanisms and injury patterns.

**Results:**

Spatial analysis revealed a cluster with a high incidence of motorbike injuries resulting in fractures. Examination of the spot demonstrated the risky combination of a speed breaker and a broken traffic light. After fixing the light, no further accidents occurred at the site.

**Conclusion:**

GIS is a promising technology for geo-referencing accident data, and may be a valuable tool to identify areas of priority for injury prevention in India.

## Background

Road traffic injuries belong to the ten leading causes of death and disability worldwide, and have emerged as a serious public health concern [1].

Identifying human, technical, and environmental factors that contribute to the incidence and severity of accidents and their health-related consequences is mandatory to establish effective prevention strategies [2-4].

GIS is the abbreviation for geographic information system. Originally developed for urban and facilities management [5,6], GIS depicts and analyzes the spatial features of, and the location and chronology of events occurring in the area of interest [7].

Innovative cartography allows for three-dimensional reconstruction of the scene. Complex environmental data are separated into their principle components, which are subsequently merged for final analysis.

In this exploratory study, we set out to describe the spatial distribution of road crashes in a community in India, and to analyze environmental factors responsible for clustering of injury events.

## Methods

This study was conducted at a trauma center located at the national highway number 7 in India. We retrospectively identified all injured subjects who had been referred to our department after sustaining a road traffic accident between April 2000 and September 2000. All patients were, at least, diagnosed with a long bone fracture. For this study, we extracted age, gender, accident mechanisms and causes, and vehicle types from our trauma database.

A set of digital thematic maps was generated from the non-scaled patwari map (local village map). Vector spaces were spanned over these maps using GIS software (JT Maps, Jlets Technology, Noida, India), and data from the identified trauma cases were added. Spatial analysis and overlay tools were used to identify local clusters of events. Clusters included all events occurring within a 50 m perimeter around a reference point. Color coding was used to mark accident and injury characteristics among clusters.

## Results

A total of 165 trauma cases were recorded during the study period. There were 111 men and 54 women, with a mean age of 37.2 ± 15.3 years (range 10 – 89 years). Crashes involved 32 bicycles (19.4%), 86 motorbikes (52.1%), and 47 cars (28.5%). Overturns were recorded in 17 cases (10.3%).

Apart from fractures, 85 subjects (51.5%) had encountered head injuries. Twenty-eight drivers were drunk (17.2%).

**Figure 1:**
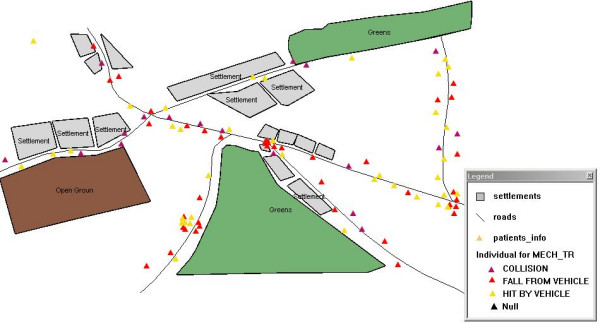
GIS-map showing the distribution of road traffic accidents in the investigated region.

The distribution of accidents along the surveyed roads is illustrated in Figure 1.

**Figure 2:**
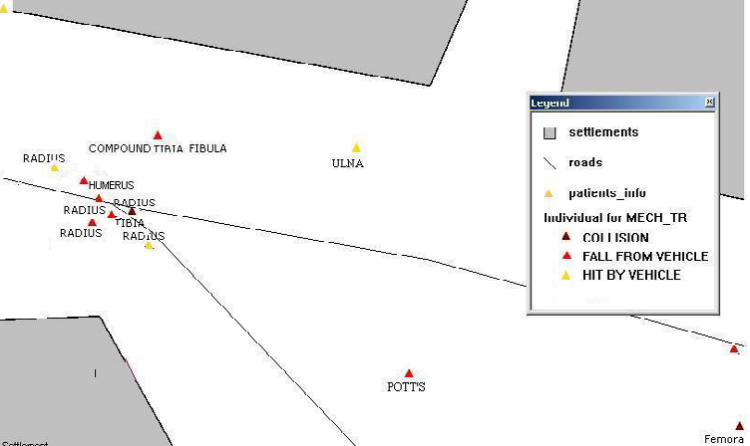
A cluster of motorbike accidents identified at a site with a speed breaker and a broken traffic light.

A detailed analysis revealed a cluster with a disproportionately high number of fracture cases (4 forearm fractures, 1 fracture of the humerus, and another fracture of the tibia, see Figure 2). All subjects who had sustained an injury in this cluster were males between 20 to 35 years of age, and all had been riding a motorbike. Physical inspection of the area revealed the presence of a speed breaker, but a broken overhead light.

It was retrospectively confirmed that all the accidents had occurred during evening and night, when the illumination was deficient. Once the problems had been fixed, no accidents occurred at that site during the next six months

For additional data see Additional file 1.

## Discussion

India contributes to about 10% of the global death toll caused by road crashes. However, it is assumed that only 0.4 out of 1.4 million serious traffic accidents are recorded and scientifically investigated, thus underestimating the burden to the health care system.

The use of maps in medical research dates back to 1854, when John Snow traced the spread of cholera to the pump at the corner of Cambridge and Broad Street in London. This classic work stresses the value of spatial analysis for the understanding and solution of public health problems.

GIS is now extensively used in different areas of health care. Epidemiologists use GIS to study health hazards in the community, to develop policies and plans for prevention on both an individual and population-based level, and to overcome inequities in health care delivery [8-12].

This study suggests some potential for the use of GIS in trauma research, especially in rural areas with limited resources. By revealing a distinct accident cluster, the most likely risk factor for future events (i.e., a broken traffic light) could effectively be eliminated.

Most accidents on Indian highways tend to occur at sites with poor road design features. Although we used only a basic set of demographic and injury-related items, and the consequences of spatial analysis were exemplified by a single scenario, we feel that GIS is an innovative approach to overcome weaknesses in the planning and maintenance of Indian roadways, and to improve traffic control and safety measures [13].

Shortening the interval to basic life support, and rapid access to comprehensive trauma care within the so-called "golden hour of shock" is crucial for the survival of trauma victims. Identifying areas with a high incidence of traffic accidents may allow for the strategic positioning of response vehicles, thereby minimizing arrival times of rescue teams on scene and transferal times of patients to hospitals. Also, information obtained by spatial analysis may help to tailor the implementation and structure of trauma centers in rural and urban settings to the expected needs and volumes [14-16].

## Conclusion

GIS is a promising tool to collect and analyze geographic and environmental data on road traffic crashes. Spatial analysis may help to identify regions of a particular high risk for traffic accidents, and to facilitate effective measures of primary and secondary prevention.

## Supplementary Material

Additional file 1 master chart Click here for file
