# Supplementary material for: A geographic information system to study trauma epidemiology in India
Source: J Trauma Manag Outcomes. 2007 Nov 26;1:3. doi: 10.1186/1752-2897-1-3 (PMC2241765; doi:10.1186/1752-2897-1-3)
Supplement: Additional file 1 — master chart [file 1752-2897-1-3-S1.doc]

| | Case ID | | --- | | 496 | | 498 | | 501 | | 511 | | 523 | | 544 | | 555 | | 557 | | 568 | | 569 | | 573 | | 574 | | 580 | | 589 | | 601 | | 603 | | 604 | | 611 | | 615 | | 621 | | 629 | | 632 | | 636 | | 640 | | 644 | | 647 | | 650 | | 658 | | 659 | | 660 | | 666 | | 669 | | 681 | | 694 | | 697 | | 702 | | 711 | | 714 | | 720 | | 723 | | 726 | | 740 | | 746 | | 748 | | 750 | | 756 | | 758 | | 760 | | 764 | | 768 | | 770 | | 772 | | 774 | | 777 | | 779 | | 793 | | 794 | | 798 | | 802 | | 806 | | 809 | | 814 | | 820 | | 835 | | 887 | | 889 | | 893 | | 897 | | 916 | | 924 | | 927 | | 934 | | 954 | | 955 | | 967 | | 988 | | 994 | | 998 | | 1003 | | 1007 | | 1009 | | 1012 | | 1017 | | 1019 | | 1024 | | 1027 | | 108 | | 1029 | | 1035 | | 1040 | | 1044 | | 1048 | | 1052 | | 1057 | | 1058 | | 1067 | | 1078 | | 1085 | | 1093 | | 1118 | | 1134 | | 1137 | | 1154 | | 1159 | | 1172 | | 1185 | | 1189 | | 1196 | | 1210 | | 1215 | | 1224 | | 1226 | | 1232 | | 1235 | | 1238 | | 1334 | | 1337 | | 1339 | | 1347 | | 1354 | | 1358 | | 1360 | | 1372 | | 1374 | | 1380 | | 1382 | | 1386 | | 1390 | | 1394 | | 1397 | | 1402 | | 1412 | | 1421 | | 1432 | | 1444 | | 1453 | | 1456 | | 1473 | | 1477 | | 1479 | | 1480 | | 1489 | | 1494 | | 1512 | | 1516 | | 1520 | | 1532 | | 1539 | | 1547 | | 1563 | | 1569 | | 1574 | | 1583 | | 1594 | | 1602 | | 1611 | | 1618 | | 1634 | | 1642 | | 1647 | | 1656 | | 1670 | | 1672 | | 1677 | | 1690 | | | Age | | --- | | 23 | | 34 | | 45 | | 28 | | 36 | | 42 | | 34 | | 45 | | 66 | | 72 | | 35 | | 13 | | 26 | | 66 | | 37 | | 81 | | 28 | | 19 | | 34 | | 37 | | 54 | | 36 | | 33 | | 28 | | 33 | | 26 | | 56 | | 62 | | 19 | | 24 | | 34 | | 56 | | 21 | | 28 | | 48 | | 36 | | 49 | | 51 | | 29 | | 67 | | 26 | | 48 | | 33 | | 12 | | 20 | | 37 | | 10 | | 81 | | 38 | | 27 | | 39 | | 41 | | 11 | | 68 | | 73 | | 27 | | 29 | | 31 | | 26 | | 33 | | 21 | | 27 | | 37 | | 33 | | 21 | | 56 | | 42 | | 27 | | 51 | | 33 | | 26 | | 47 | | 43 | | 33 | | 19 | | 47 | | 51 | | 21 | | 25 | | 85 | | 47 | | 32 | | 28 | | 26 | | 19 | | 67 | | 44 | | 38 | | 27 | | 51 | | 48 | | 23 | | 24 | | 43 | | 29 | | 28 | | 15 | | 34 | | 31 | | 29 | | 30 | | 30 | | 31 | | 44 | | 22 | | 23 | | 47 | | 38 | | 49 | | 56 | | 57 | | 23 | | 17 | | 35 | | 40 | | 57 | | 45 | | 66 | | 33 | | 29 | | 44 | | 28 | | 89 | | 45 | | 67 | | 23 | | 37 | | 32 | | 33 | | 67 | | 45 | | 37 | | 38 | | 26 | | 25 | | 38 | | 23 | | 16 | | 29 | | 26 | | 45 | | 37 | | 29 | | 44 | | 37 | | 30 | | 27 | | 20 | | 30 | | 45 | | 36 | | 37 | | 28 | | 26 | | 28 | | 33 | | 31 | | 32 | | 56 | | 57 | | 32 | | 24 | | 28 | | 54 | | 23 | | | Sex | | --- | | m | | m | | m | | m | | f | | m | | m | | m | | m | | f | | f | | f | | m | | m | | f | | m | | m | | m | | m | | m | | m | | f | | f | | m | | f | | m | | m | | m | | m | | m | | m | | m | | f | | f | | m | | f | | f | | m | | f | | m | | f | | m | | m | | m | | f | | m | | m | | f | | m | | m | | m | | m | | m | | m | | m | | m | | m | | f | | f | | m | | m | | m | | m | | m | | f | | m | | f | | m | | f | | m | | m | | m | | f | | f | | f | | m | | f | | m | | f | | m | | f | | m | | m | | m | | m | | m | | f | | f | | m | | f | | f | | f | | f | | m | | f | | m | | m | | m | | m | | m | | f | | m | | m | | f | | m | | m | | m | | f | | m | | f | | m | | f | | m | | f | | m | | m | | m | | m | | m | | f | | f | | m | | m | | f | | m | | m | | m | | f | | m | | m | | m | | f | | m | | m | | m | | f | | m | | f | | m | | f | | m | | f | | m | | f | | m | | f | | m | | f | | m | | m | | m | | m | | f | | m | | m | | m | | m | | f | | m | | m | | m | | m | | m | | m | | m | | | Date of injury | | --- | | 1.4 | | 1.4 | | 3.4 | | 4.4 | | 5.4 | | 5.4 | | 5.4 | | 6.4 | | 8.4 | | 9.4 | | 11.4 | | 12.4 | | 15.4 | | 17.4 | | 20.4 | | 20.4 | | 20.4 | | 21.4 | | 21.4 | | 22.4 | | 24.4 | | 25.4 | | 28.4 | | 29.4 | | 30.4 | | 1.5 | | 1.5 | | 3.5 | | 3.5 | | 3.5 | | 4.5 | | 5.5 | | 8.5 | | 10.5 | | 10.5 | | 11.5 | | 12.5 | | 13.5 | | 14.5 | | 15.5 | | 15.5 | | 17.5 | | 18.5 | | 19.5 | | 19.5 | | 20.5 | | 20.5 | | 21.5 | | 22.5 | | 24.5 | | 25.5 | | 26.5 | | 27.5 | | 29.5 | | 29.5 | | 31.5 | | 31.5 | | 1.6 | | 1.6 | | 2.6 | | 3.6 | | 3.6 | | 5.6 | | 6.6 | | 7.6 | | 7.6 | | 9.6 | | 10.6 | | 11.6 | | 12.6 | | 13.6 | | 15.6 | | 17.6 | | 18.6 | | 19.6 | | 20.6 | | 21.6 | | 22.6 | | 23.6 | | 24.6 | | 25.6 | | 26.6 | | 27.6 | | 29.6 | | 30.6 | | 1.7 | | 3.7 | | 5.7 | | 5.7 | | 6.7 | | 8.7 | | 9.7 | | 10.7 | | 11.7 | | 12.7 | | 13.7 | | 14.7 | | 15.7 | | 16.7 | | 19.7 | | 20.7 | | 21.7 | | 22.7 | | 23.7 | | 25.7 | | 26.7 | | 27.7 | | 28.7 | | 29.7 | | 30.7 | | 31.7 | | 1.8 | | 2.8 | | 4.8 | | 5.8 | | 6.8 | | 7.8 | | 10.8 | | 11.8 | | 11.8 | | 13.8 | | 14.8 | | 15.8 | | 16.8 | | 18.8 | | 19.8 | | 20.8 | | 22.8 | | 23.8 | | 25.8 | | 27.8 | | 29.8 | | 30.8 | | 1.9 | | 3.9 | | 5.9 | | 6.9 | | 7.9 | | 8.9 | | 8.9 | | 9.9 | | 10.9 | | 11.9 | | 12.9 | | 13.9 | | 14.9 | | 15.9 | | 15.9 | | 15.9 | | 16.9 | | 18.9 | | 19.9 | | 20.9 | | 21.9 | | 22.9 | | 23.9 | | 24.9 | | 27.9 | | 27.9 | | 28.9 | | 29.9 | | 30.9 | | 30.9 | | 30.9 | | 30.9 | | | mode of injury | | --- | | RTA | | RTA | | RTA | | RTA | | RTA | | overturning | | RTA | | overturning | | RTA | | RTA | | RTA | | RTA | | RTA | | RTA | | RTA | | overturning | | RTA | | RTA | | RTA | | RTA | | RTA | | RTA | | RTA | | RTA | | RTA | | RTA | | RTA | | RTA | | RTA | | RTA | | RTA | | RTA | | RTA | | RTA | | overturning | | RTA | | RTA | | overturning | | RTA | | RTA | | RTA | | RTA | | RTA | | RTA | | RTA | | overturning | | RTA | | RTA | | RTA | | RTA | | RTA | | RTA | | RTA | | RTA | | RTA | | RTA | | RTA | | RTA | | RTA | | RTA | | RTA | | RTA | | RTA | | RTA | | RTA | | RTA | | RTA | | overturning | | RTA | | RTA | | RTA | | RTA | | RTA | | RTA | | RTA | | RTA | | overturning | | RTA | | RTA | | RTA | | RTA | | RTA | | RTA | | RTA | | RTA | | RTA | | RTA | | overturning | | RTA | | RTA | | RTA | | RTA | | RTA | | RTA | | overturning | | RTA | | RTA | | RTA | | RTA | | RTA | | RTA | | RTA | | RTA | | RTA | | RTA | | RTA | | RTA | | RTA | | RTA | | overturning | | RTA | | RTA | | RTA | | RTA | | RTA | | overturning | | RTA | | fall | | RTA | | RTA | | RTA | | RTA | | RTA | | RTA | | RTA | | RTA | | RTA | | RTA | | RTA | | RTA | | RTA | | RTA | | overturning | | RTA | | RTA | | RTA | | RTA | | RTA | | RTA | | RTA | | RTA | | RTA | | RTA | | RTA | | RTA | | RTA | | RTA | | RTA | | RTA | | RTA | | RTA | | overturning | | RTA | | RTA | | overturning | | overturning | | RTA | | RTA | | RTA | | RTA | | RTA | | RTA | | RTA | | RTA | | RTA | | | Vehicle involved | | --- | | 1 | | 1 | | 2 | | 1 | | 3 | | 3 | | 2 | | 3 | | 3 | | 2 | | 3 | | 2 | | 1 | | 2 | | 3 | | 3 | | 3 | | 1 | | 1 | | 3 | | 2 | | 2 | | 2 | | 1 | | 2 | | 2 | | 2 | | 2 | | 3 | | 1 | | 3 | | 2 | | 2 | | 2 | | 3 | | 1 | | 1 | | 3 | | 1 | | 2 | | 2 | | 2 | | 2 | | 2 | | 2 | | 3 | | 2 | | 2 | | 2 | | 2 | | 2 | | 2 | | 2 | | 2 | | 2 | | 1 | | 2 | | 1 | | 3 | | 2 | | 3 | | 3 | | 1 | | 2 | | 1 | | 2 | | 3 | | 3 | | 3 | | 3 | | 1 | | 1 | | 2 | | 1 | | 3 | | 1 | | 3 | | 2 | | 2 | | 2 | | 2 | | 2 | | 2 | | 2 | | 2 | | 1 | | 2 | | 3 | | 3 | | 3 | | 3 | | 3 | | 2 | | 2 | | 3 | | 1 | | 2 | | 2 | | 2 | | 1 | | 2 | | 1 | | 1 | | 3 | | 3 | | 2 | | 2 | | 2 | | 3 | | 3 | | 3 | | 2 | | 2 | | 2 | | 2 | | 3 | | 2 | | 1 | | 1 | | 1 | | 1 | | 3 | | 2 | | 1 | | 3 | | 2 | | 2 | | 2 | | 2 | | 1 | | 2 | | 3 | | 3 | | 3 | | 2 | | 2 | | 2 | | 2 | | 2 | | 2 | | 2 | | 2 | | 2 | | 2 | | 1 | | 2 | | 3 | | 3 | | 3 | | 3 | | 2 | | 3 | | 2 | | 2 | | 3 | | 3 | | 2 | | 2 | | 2 | | 2 | | 2 | | 2 | | 1 | | 2 | | 2 | | | HI | | --- | | 0 | | 0 | | 1 | | 0 | | 1 | | 1 | | 1 | | 1 | | 1 | | 0 | | 1 | | 0 | | 1 | | 1 | | 1 | | 1 | | 0 | | 0 | | 0 | | 1 | | 0 | | 1 | | 0 | | 1 | | 0 | | 1 | | 0 | | 1 | | 0 | | 1 | | 0 | | 1 | | 0 | | 1 | | 1 | | 1 | | 0 | | 1 | | 0 | | 1 | | 0 | | 1 | | 0 | | 1 | | 1 | | 1 | | 1 | | 1 | | 0 | | 0 | | 1 | | 0 | | 0 | | 0 | | 0 | | 0 | | 1 | | 1 | | 0 | | 0 | | 1 | | 0 | | 1 | | 0 | | 1 | | 0 | | 0 | | 0 | | 1 | | 0 | | 0 | | 0 | | 1 | | 0 | | 1 | | 0 | | 1 | | 0 | | 1 | | 0 | | 1 | | 0 | | 0 | | 0 | | 0 | | 1 | | 1 | | 1 | | 1 | | 1 | | 0 | | 0 | | 0 | | 0 | | 1 | | 1 | | 0 | | 1 | | 1 | | 1 | | 0 | | 0 | | 1 | | 1 | | 0 | | 1 | | 1 | | 1 | | 1 | | 1 | | 0 | | 1 | | 0 | | 0 | | 0 | | 1 | | 0 | | 1 | | 0 | | 1 | | 0 | | 1 | | 0 | | 1 | | 1 | | 1 | | 1 | | 1 | | 1 | | 0 | | 0 | | 0 | | 1 | | 0 | | 1 | | 1 | | 1 | | 0 | | 0 | | 0 | | 0 | | 1 | | 1 | | 1 | | 0 | | 0 | | 0 | | 1 | | 1 | | 1 | | 0 | | 1 | | 0 | | 1 | | 0 | | 1 | | 0 | | 0 | | 1 | | 1 | | 1 | | 0 | | 0 | | 0 | | 0 | | | Other Injuries | | --- | | 1 | | 1 | | 10 | | 0 | | 1 | | 1 | | 1 | | 1 | | 1 | | 1 | | 0 | | 0 | | 1 | | 1 | | 1 | | 1 | | 0 | | 0 | | 0 | | 1 | | 0 | | 1 | | 0 | | 1 | | 0 | | 1 | | 0 | | 1 | | 0 | | 1 | | 0 | | 1 | | 0 | | 1 | | 1 | | 1 | | 0 | | 1 | | 0 | | 1 | | 0 | | 1 | | 0 | | 1 | | 1 | | 1 | | 1 | | 1 | | 0 | | 0 | | 0 | | 0 | | 0 | | 0 | | 0 | | 0 | | 0 | | 1 | | 1 | | 1 | | 0 | | 0 | | 0 | | 0 | | 0 | | 0 | | 1 | | 1 | | 1 | | 1 | | 0 | | 0 | | 0 | | 0 | | 1 | | 0 | | 0 | | 0 | | 0 | | 1 | | 1 | | 1 | | 1 | | 0 | | 0 | | 1 | | 1 | | 0 | | 0 | | 0 | | 1 | | 1 | | 1 | | 0 | | 0 | | 0 | | 0 | | 1 | | 0 | | 1 | | 0 | | 1 | | 1 | | 1 | | 0 | | 1 | | 1 | | 1 | | 1 | | 1 | | 0 | | 1 | | 0 | | 0 | | 0 | | 1 | | 0 | | 1 | | 0 | | 1 | | 0 | | 1 | | 0 | | 1 | | 1 | | 1 | | 1 | | 1 | | 1 | | 0 | | 0 | | 0 | | 1 | | 0 | | 1 | | 1 | | 0 | | 1 | | 1 | | 1 | | 1 | | 1 | | 1 | | 1 | | 0 | | 0 | | 0 | | 1 | | 1 | | 1 | | 0 | | 1 | | 0 | | 1 | | 0 | | 1 | | 0 | | 0 | | 1 | | 1 | | 1 | | 0 | | 0 | | 0 | | 0 | | | Alchol | | --- | | 0 | | 0 | | 0 | | 0 | | 0 | | 0 | | 0 | | 0 | | 1 | | 0 | | 0 | | 1 | | 0 | | 0 | | 0 | | 0 | | 0 | | 0 | | 0 | | 0 | | 0 | | 0 | | 0 | | 1 | | 0 | | 0 | | 0 | | 1 | | 0 | | 0 | | 0 | | 0 | | 0 | | 0 | | 0 | | 0 | | 0 | | 0 | | 0 | | 0 | | 0 | | 1 | | 0 | | 0 | | 0 | | 0 | | 0 | | 0 | | 0 | | 0 | | 0 | | 1 | | 0 | | 0 | | 0 | | 0 | | 0 | | 0 | | 0 | | 0 | | 1 | | 0 | | 0 | | 1 | | 1 | | 1 | | 0 | | 0 | | 0 | | 0 | | 0 | | 0 | | 1 | | 1 | | 1 | | 0 | | 0 | | 0 | | 0 | | 0 | | 0 | | 0 | | 0 | | 0 | | 0 | | 0 | | 0 | | 0 | | 0 | | 1 | |  | | 0 | | 0 | | 0 | | 0 | | 0 | | 0 | | 0 | | 0 | | 0 | | 1 | |  | | 0 | | 1 | | 0 | | 0 | | 0 | | 0 | | 1 | | 1 | | 1 | | 0 | | 0 | | 0 | | 1 | | 1 | | 0 | | 0 | | 0 | | 0 | | 0 | | 0 | | 0 | | 0 | | 0 | | 0 | | 0 | | 0 | | 0 | | 0 | | 1 | | 0 | | 0 | | 0 | | 0 | | 0 | | 0 | | 0 | | 0 | | 0 | | 0 | | 0 | | 0 | | 0 | | 0 | | 0 | | 1 | | 1 | | 1 | | 1 | | 0 | | 0 | | 0 | | 0 | | 0 | | 0 | | 0 | | 0 | | 0 | | 0 | | 0 | | 0 | | 1 | | 1 | | 0 | |
| --- | --- | --- | --- | --- | --- | --- | --- | --- | --- | --- | --- | --- | --- | --- | --- | --- | --- | --- | --- | --- | --- | --- | --- | --- | --- | --- | --- | --- | --- | --- | --- | --- | --- | --- | --- | --- | --- | --- | --- | --- | --- | --- | --- | --- | --- | --- | --- | --- | --- | --- | --- | --- | --- | --- | --- | --- | --- | --- | --- | --- | --- | --- | --- | --- | --- | --- | --- | --- | --- | --- | --- | --- | --- | --- | --- | --- | --- | --- | --- | --- | --- | --- | --- | --- | --- | --- | --- | --- | --- | --- | --- | --- | --- | --- | --- | --- | --- | --- | --- | --- | --- | --- | --- | --- | --- | --- | --- | --- | --- | --- | --- | --- | --- | --- | --- | --- | --- | --- | --- | --- | --- | --- | --- | --- | --- | --- | --- | --- | --- | --- | --- | --- | --- | --- | --- | --- | --- | --- | --- | --- | --- | --- | --- | --- | --- | --- | --- | --- | --- | --- | --- | --- | --- | --- | --- | --- | --- | --- | --- | --- | --- | --- | --- | --- | --- | --- | --- | --- | --- | --- | --- | --- | --- | --- | --- | --- | --- | --- | --- | --- | --- | --- | --- | --- | --- | --- | --- | --- | --- | --- | --- | --- | --- | --- | --- | --- | --- | --- | --- | --- | --- | --- | --- | --- | --- | --- | --- | --- | --- | --- | --- | --- | --- | --- | --- | --- | --- | --- | --- | --- | --- | --- | --- | --- | --- | --- | --- | --- | --- | --- | --- | --- | --- | --- | --- | --- | --- | --- | --- | --- | --- | --- | --- | --- | --- | --- | --- | --- | --- | --- | --- | --- | --- | --- | --- | --- | --- | --- | --- | --- | --- | --- | --- | --- | --- | --- | --- | --- | --- | --- | --- | --- | --- | --- | --- | --- | --- | --- | --- | --- | --- | --- | --- | --- | --- | --- | --- | --- | --- | --- | --- | --- | --- | --- | --- | --- | --- | --- | --- | --- | --- | --- | --- | --- | --- | --- | --- | --- | --- | --- | --- | --- | --- | --- | --- | --- | --- | --- | --- | --- | --- | --- | --- | --- | --- | --- | --- | --- | --- | --- | --- | --- | --- | --- | --- | --- | --- | --- | --- | --- | --- | --- | --- | --- | --- | --- | --- | --- | --- | --- | --- | --- | --- | --- | --- | --- | --- | --- | --- | --- | --- | --- | --- | --- | --- | --- | --- | --- | --- | --- | --- | --- | --- | --- | --- | --- | --- | --- | --- | --- | --- | --- | --- | --- | --- | --- | --- | --- | --- | --- | --- | --- | --- | --- | --- | --- | --- | --- | --- | --- | --- | --- | --- | --- | --- | --- | --- | --- | --- | --- | --- | --- | --- | --- | --- | --- | --- | --- | --- | --- | --- | --- | --- | --- | --- | --- | --- | --- | --- | --- | --- | --- | --- | --- | --- | --- | --- | --- | --- | --- | --- | --- | --- | --- | --- | --- | --- | --- | --- | --- | --- | --- | --- | --- | --- | --- | --- | --- | --- | --- | --- | --- | --- | --- | --- | --- | --- | --- | --- | --- | --- | --- | --- | --- | --- | --- | --- | --- | --- | --- | --- | --- | --- | --- | --- | --- | --- | --- | --- | --- | --- | --- | --- | --- | --- | --- | --- | --- | --- | --- | --- | --- | --- | --- | --- | --- | --- | --- | --- | --- | --- | --- | --- | --- | --- | --- | --- | --- | --- | --- | --- | --- | --- | --- | --- | --- | --- | --- | --- | --- | --- | --- | --- | --- | --- | --- | --- | --- | --- | --- | --- | --- | --- | --- | --- | --- | --- | --- | --- | --- | --- | --- | --- | --- | --- | --- | --- | --- | --- | --- | --- | --- | --- | --- | --- | --- | --- | --- | --- | --- | --- | --- | --- | --- | --- | --- | --- | --- | --- | --- | --- | --- | --- | --- | --- | --- | --- | --- | --- | --- | --- | --- | --- | --- | --- | --- | --- | --- | --- | --- | --- | --- | --- | --- | --- | --- | --- | --- | --- | --- | --- | --- | --- | --- | --- | --- | --- | --- | --- | --- | --- | --- | --- | --- | --- | --- | --- | --- | --- | --- | --- | --- | --- | --- | --- | --- | --- | --- | --- | --- | --- | --- | --- | --- | --- | --- | --- | --- | --- | --- | --- | --- | --- | --- | --- | --- | --- | --- | --- | --- | --- | --- | --- | --- | --- | --- | --- | --- | --- | --- | --- | --- | --- | --- | --- | --- | --- | --- | --- | --- | --- | --- | --- | --- | --- | --- | --- | --- | --- | --- | --- | --- | --- | --- | --- | --- | --- | --- | --- | --- | --- | --- | --- | --- | --- | --- | --- | --- | --- | --- | --- | --- | --- | --- | --- | --- | --- | --- | --- | --- | --- | --- | --- | --- | --- | --- | --- | --- | --- | --- | --- | --- | --- | --- | --- | --- | --- | --- | --- | --- | --- | --- | --- | --- | --- | --- | --- | --- | --- | --- | --- | --- | --- | --- | --- | --- | --- | --- | --- | --- | --- | --- | --- | --- | --- | --- | --- | --- | --- | --- | --- | --- | --- | --- | --- | --- | --- | --- | --- | --- | --- | --- | --- | --- | --- | --- | --- | --- | --- | --- | --- | --- | --- | --- | --- | --- | --- | --- | --- | --- | --- | --- | --- | --- | --- | --- | --- | --- | --- | --- | --- | --- | --- | --- | --- | --- | --- | --- | --- | --- | --- | --- | --- | --- | --- | --- | --- | --- | --- | --- | --- | --- | --- | --- | --- | --- | --- | --- | --- | --- | --- | --- | --- | --- | --- | --- | --- | --- | --- | --- | --- | --- | --- | --- | --- | --- | --- | --- | --- | --- | --- | --- | --- | --- | --- | --- | --- | --- | --- | --- | --- | --- | --- | --- | --- | --- | --- | --- | --- | --- | --- | --- | --- | --- | --- | --- | --- | --- | --- | --- | --- | --- | --- | --- | --- | --- | --- | --- | --- | --- | --- | --- | --- | --- | --- | --- | --- | --- | --- | --- | --- | --- | --- | --- | --- | --- | --- | --- | --- | --- | --- | --- | --- | --- | --- | --- | --- | --- | --- | --- | --- | --- | --- | --- | --- | --- | --- | --- | --- | --- | --- | --- | --- | --- | --- | --- | --- | --- | --- | --- | --- | --- | --- | --- | --- | --- | --- | --- | --- | --- | --- | --- | --- | --- | --- | --- | --- | --- | --- | --- | --- | --- | --- | --- | --- | --- | --- | --- | --- | --- | --- | --- | --- | --- | --- | --- | --- | --- | --- | --- | --- | --- | --- | --- | --- | --- | --- | --- | --- | --- | --- | --- | --- | --- | --- | --- | --- | --- | --- | --- | --- | --- | --- | --- | --- | --- | --- | --- | --- | --- | --- | --- | --- | --- | --- | --- | --- | --- | --- | --- | --- | --- | --- | --- | --- | --- | --- | --- | --- | --- | --- | --- | --- | --- | --- | --- | --- | --- | --- | --- | --- | --- | --- | --- | --- | --- | --- | --- | --- | --- | --- | --- | --- | --- | --- | --- | --- | --- | --- | --- | --- | --- | --- | --- | --- | --- | --- | --- | --- | --- | --- | --- | --- | --- | --- | --- | --- | --- | --- | --- | --- | --- | --- | --- | --- | --- | --- | --- | --- | --- | --- | --- | --- | --- | --- | --- | --- | --- | --- | --- | --- | --- | --- | --- | --- | --- | --- | --- | --- | --- | --- | --- | --- | --- | --- | --- | --- | --- | --- | --- | --- | --- | --- | --- | --- | --- | --- | --- | --- | --- | --- | --- | --- | --- | --- | --- | --- | --- | --- | --- | --- | --- | --- | --- | --- | --- | --- | --- | --- | --- | --- | --- | --- | --- | --- | --- | --- | --- | --- | --- | --- | --- | --- | --- | --- | --- | --- | --- | --- | --- | --- | --- | --- | --- | --- | --- | --- | --- | --- | --- | --- | --- | --- | --- | --- | --- | --- | --- | --- | --- | --- | --- | --- | --- | --- | --- | --- | --- | --- | --- | --- | --- | --- | --- | --- | --- | --- | --- | --- | --- | --- | --- | --- | --- | --- | --- | --- | --- | --- | --- | --- | --- | --- | --- | --- | --- | --- | --- | --- | --- | --- | --- | --- | --- | --- | --- | --- | --- | --- | --- | --- | --- | --- | --- | --- | --- | --- | --- | --- | --- | --- | --- | --- | --- | --- | --- | --- | --- | --- | --- | --- | --- | --- | --- | --- | --- | --- | --- | --- | --- | --- | --- | --- | --- | --- | --- | --- | --- | --- | --- | --- | --- | --- | --- | --- | --- | --- | --- | --- | --- | --- | --- | --- | --- | --- | --- | --- | --- | --- | --- | --- | --- | --- | --- | --- | --- | --- | --- | --- | --- | --- | --- | --- | --- | --- | --- | --- | --- | --- | --- | --- | --- | --- | --- | --- | --- | --- | --- | --- | --- | --- | --- | --- | --- | --- | --- | --- | --- | --- | --- | --- | --- | --- | --- | --- | --- | --- | --- | --- | --- | --- | --- | --- | --- | --- | --- | --- | --- | --- | --- | --- | --- | --- | --- | --- | --- | --- | --- | --- | --- | --- | --- | --- | --- | --- | --- | --- | --- | --- | --- | --- | --- | --- | --- | --- | --- | --- | --- | --- | --- | --- | --- | --- | --- | --- | --- | --- | --- | --- | --- | --- | --- | --- | --- | --- | --- | --- | --- | --- | --- | --- | --- | --- | --- | --- | --- | --- | --- | --- | --- | --- | --- | --- | --- | --- | --- | --- | --- | --- | --- | --- | --- | --- | --- | --- | --- | --- | --- | --- | --- | --- | --- | --- | --- | --- | --- | --- | --- | --- | --- | --- | --- | --- | --- | --- | --- | --- | --- | --- | --- | --- | --- | --- | --- | --- | --- | --- | --- | --- | --- | --- | --- | --- | --- | --- | --- | --- | --- | --- | --- | --- | --- | --- | --- | --- | --- | --- | --- | --- | --- | --- | --- |

Legends:

RTA: Road Traffic Accident.

Vehicle Incolved:

0 – Paedistrian

1 – Two wheeler, non motor driven

2- Motor Driven two wheeler

3 – Four wheeler.

For head injury/other injury/Alchol:

0: The said factor was absent

1: the factor was present.
